# Commentator Discussion: Malnutrition in adult patients treated with venoarterial membrane oxygenation: A descriptive cohort study

**DOI:** 10.1016/j.xjon.2024.10.011

**Published:** 2024-10-18

See Article page 38.

Presenter: Stacy Pelekhaty

**Unidentified Speaker 1**. Our commentator will be Dr Hackmann.

**Dr Amy Hackmann***(Boston, Mass)*. Thank you, Ms Pelekhaty and the whole University of Maryland team for presenting this work, and thank you for providing your manuscript in advance. Really, this is an outstanding evaluation of nearly 200 patients on VA-ECMO, their average time on VA-ECMO of about 6 days, and 65% of those patients survived, which in itself is remarkable. So really, congrats to your team. Unfortunately, you identified that half of those patients had malnutrition. A third of them came to the hospital without a problem, but the other two-thirds developed it in the hospital. And this is something I think we all see on rounds in the ICU—that patients, their two feeds get held today for a CAT scan and tomorrow for a line in the cath lab and them tomorrow, for another procedure—and so really, how we get behind very easily in taking care of these patients. Then we can see how this has a negative effect on the patient, their time in the hospital, their time on ECMO, and their survival, about a 20% difference in survival. So really, thank you for that.

**Figure:**
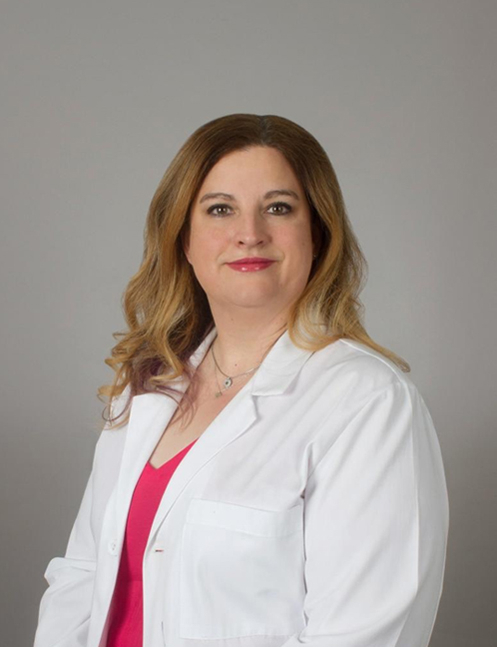

I do have a few questions for you. Really, how can we do better to avoid this in our patients? How do we keep them from developing this malnutrition? Are there any biomarkers that, taken together, can help us identify this? Are there any imaging studies, whether it be pectoralis muscle thickness, soleus thickness, anything we can do that we can regularly evaluate our patients and hopefully, not get behind in those patients that develop malnutrition in the hospital on ECMO?

**Stacy Pelekhaty***(Baltimore, Md)*. The short answer is, unfortunately, we don't know as much as I would hope we would at this point. This year marks the 50-year anniversary of the publication of the [Butterworth Nutrition Today 9(2):p 4-8, March 1974] article, “Malnutrition, the Skeleton in the Hospital Closet,” and we still are behind the eight-ball about being able to identify and prevent hospital-acquired malnutrition. Some of the challenges are what you mentioned. We call that affectionately “starvation through optimism,” that we know our patients are not going to get 100% of their goal, and we are resistant to starting early parental nutrition for a range of reasons. Some of the things that we know can help are starting nutrition support early, being on top of whether or not our patients are receiving that, and that is where your dietitian can really be your best friend. If your dietitian is not rounding with you in the ICU, talk to your dietitian about what can happen to get them there. If your dietitian isn't explaining to you how your patients are being fed or not being fed, ask them how you can get them to bring that information to rounds. Oftentimes we have this information, and dietitians just don't know whether or not it's okay for them to speak up. We're often told that we need to be invited to the table before we can talk. So, I think that's a big piece of it.

**Figure:**
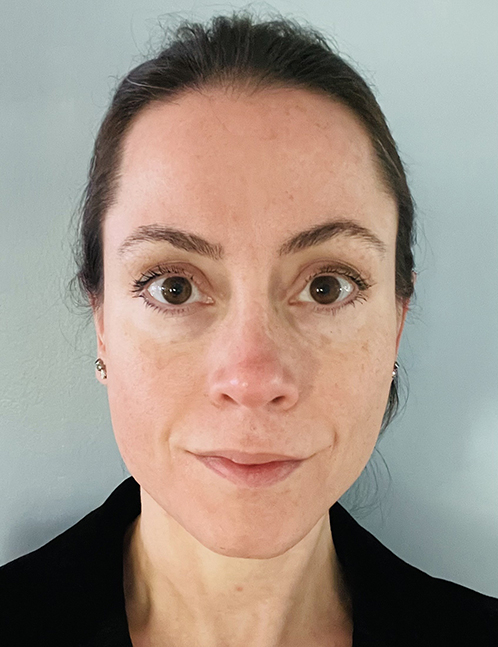

To the point of how we can prevent hospital-acquired malnutrition and how biomarkers play into that, we know that inflammatory metabolism is a big component in developing hospital-acquired malnutrition when we take out the starvation component of it. Things like CRP and IL-6, monitoring them can tell you whether or not your patient has inflammatory metabolism as a component of that. If you know that information, however, we don't necessarily know how to best feed our patients, and that is, I think, the next step, and some of our future research efforts in better determining how we can move the needle forward in preventing hospital-acquired malnutrition.

**Unidentified Speaker 1**. Thank you.

**Dr Hackmann**. My next question is that you have a relatively high use of TPN in these patients, almost 20%, which I know has not been my experience in taking care of ECMO patients. Was there a lot of resistance at your center to getting people to use TPN, because I know there's a lot of concern especially about infection with TPN.

**Ms Pelekhaty**. Yeah, there definitely is. And a lot of it is, “why would we start TPN today when we can, hopefully, just feed them enterally tomorrow?” And the infection question definitely comes up. A lot of the large, randomized control trials that have been done recently, particularly the NUTRIREA-2 study that was done in France in 2013, have showed that when comparing early enteral to early parenteral nutrition in very high-risk populations, there was no difference in infectious complications between the 2 cohorts.

**Dr Hackmann**. And then I just want to give you one more chance to reiterate your data on ECMO time is longer, ICU time is longer, discharge to rehab [inaudible] is more, and death is higher in patients that are malnourished.

**Ms Pelekhaty**. Yeah, and unfortunately, we don't have the information on discharge destination in our population, but that is definitely something that we would like to look into in the future. But you are absolutely right. Our patients were on ECMO longer, which means they're in the hospital longer, and our mortality rates were higher.

**Unidentified Speaker 1**. Thank you so much. It looks like we have two comments and questions.

**Dr Hackmann**. Congrats on totally being the only dietician here.

**Ms Pelekhaty**. [laughter] Thank you so much.

**Unidentified Speaker 2**. Stacy, that was a fabulous talk. I want to point out to everybody this issue of developing malnutrition while on ECMO. Oxygen consumption and CO_2_ production are the 2 molecules that determine your caloric need. Those 2 molecules are it. And to the degree that the ECMO circuit can tell everybody how much oxygen this person is getting per minute, and it can, and how much CO_2_ are they eliminating per minute, and it can by looking at the CO_2_ coming out of the bottom of the oxygenator, you now have the 2 variables that tell you what the caloric needs of that patient are. And when you compare it to what the ASPEN thinks with their guidelines, they can be off by as much as 100%. So just keep that in mind, in terms of helping to guide how much calories you need to give a person a day.

**Ms Pelekhaty**. Very, very briefly, I'll share that we've developed a novel method for incorporating indirect calorimetry in the population. As I was preparing to come to this conference and was prepping my dieticians, I was summarizing some of our observational data, which we hope to be presenting later this year, and our cals per kilo range was 6 to 40 k/cals per kilo per day in the population. So it is very, very difficult to guess right in ECMO patients, in terms of what they truly need.

**Unidentified Speaker 1**. Wow, I'll look forward to that. Fascinating.

**Ms Pelekhaty**. Thank you so much.

## Conflict of Interest Statement

The authors reported no conflicts of interest.

The *Journal* policy requires editors and reviewers to disclose conflicts of interest and to decline handling or reviewing manuscripts for which they may have a conflict of interest. The editors and reviewers of this article have no conflicts of interest.

